# Cortical modulation of neuronal activity in the cat's lateral geniculate and perigeniculate nuclei: a modeling study

**DOI:** 10.1186/1471-2202-12-S1-P373

**Published:** 2011-07-18

**Authors:** Jacek Rogala, Wioletta Waleszczyk, Andrzej Wróbel, Daniel K Wójcik

**Affiliations:** 1 Laboratory of Neuroinformatics, Dept. of Neurophysiology, Nencki Institute, 02-093 Warsaw, Poland; 2 Laboratory of the Visual System, Dept. of Neurophysiology, Nencki Institute, 02-093 Warsaw, Poland

## 

The role of cortical feedback in thalamo-cortical processing loop have been extensively investigated over the last decades. In general, these studies focused on cortical feedback exerted over lateral geniculate nucleus (LGN) principal cells, only in several cases the effects of cortical inactivation were investigated simultaneously in both thalamic relay cells and perigeniculate nucleus (PGN) inhibitory neurons. In the previous study [1] we showed in the cat that cessation of cortical input by cooling of visual cortex (areas 17 and 18) decreased spontaneous activity of LGN relay cells and increased spontaneous activity of PGN neurons. In contrast, visually evoked responses of most PGN neurons and LGN principal cells both decreased.

To identify network mechanisms underlying such functional changes we conducted a modelling study in NEURON on several networks of point neurons with varied model parameters, such as membrane properties, synaptic weights and axonal delays. We considered five network topologies of the retino-geniculo-cortical pathway [2-6].

**Figure 1:**
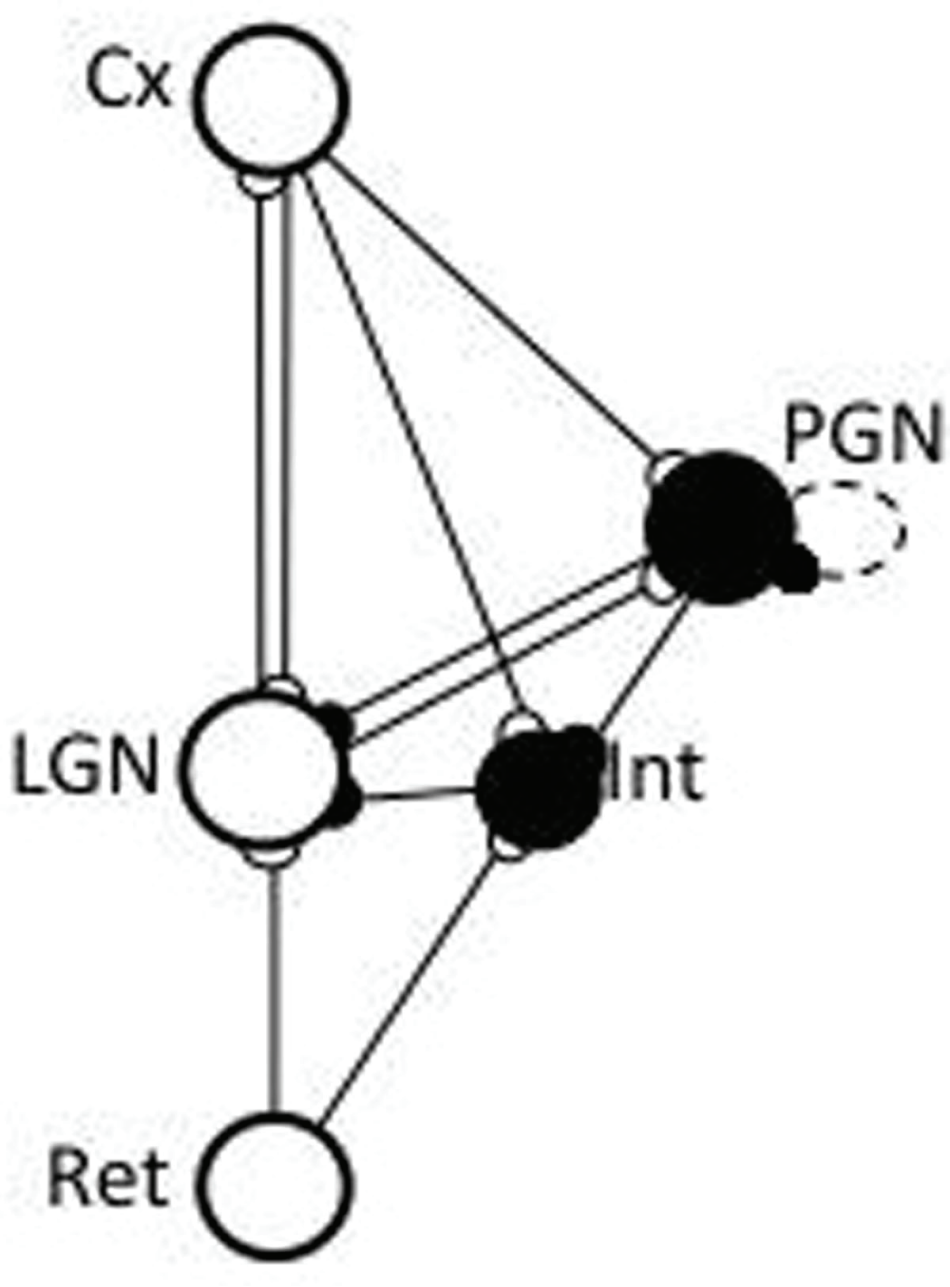
The model network of the thalamo-cortical loop following [2]. The model in [3] does not include LGN feedforward interneuron. Both variants reproduced experimental results [1]. White circles – relay cells, black – inhibitory neurons.

All models were robust against changes of axonal delays except for delay between LGN feed-forward interneuron and principal cell when this connection was present. In all such cases the models were found to be very sensitive to this delay. To reproduce the experimental results [1] the models required: 1) reciprocally connected PGN cells, connection present only in two model variants [2,3], and 2) slow decay of intracellular calcium. We found model [2] most consistent with known physiology and anatomy of the cat. Table 1. Figure 1.

**Table 1:** Mean spontaneous activity (spikes/s) for tested models (mean of 30 repetitions) and *t*-test P values.

	LGN relay model cell			PGN model cell		
	
Model from paper	Control	Cortical cooling	P	Control	Cortical cooling	P
[2]	**18.73**	**16.4**	**0**	**9.27**	**15.83**	**0**
[3]	25.33	21.27	0	8.57	19.77	0
[4]	18.97	20.2	0.14	6.57	7.93	0
[5]	17.73	20.2	0	7.93	7.93	1
[6]	20.93	24.97	0	-	-	-
